# What have the social sciences ever done for equity in health policy and health systems?

**DOI:** 10.1186/s12939-018-0842-9

**Published:** 2018-09-24

**Authors:** Trisha Greenhalgh

**Affiliations:** Nuffield Department of Primary Care Health Sciences, University of Oxford, Oxford, UK

**Keywords:** Social sciences, Health equity, Narrative

## Abstract

**Background:**

The social sciences can be defined as the scientific study of human society and social relationships.

**Main text:**

A number of underpinning disciplines within the social sciences, notably sociology, social psychology and anthropology, as well as interdisciplinary fields like science and technology studies and migration studies, offer both theoretical insights and methodological approaches which can productively enhance the study of equity in health systems and policy research. In particular, qualitative research in general and the use of narrative methods in particular can help illuminate individual experience and the interaction of multiple structural influences on that experience.

**Conclusion:**

This article sets the theoretical scene for a special issue of the journal on social sciences and equity.

The social sciences can be defined as the scientific study of human society and social relationships. They include the disciplines of sociology, social psychology, anthropology, social policy, human geography, political science and economics – as well as interdisciplinary fields such migration studies, science and technology studies and global health. These disciplines and fields cover a host of potential research topics and questions – and also a wide range of underpinning philosophical assumptions about the nature of social reality (ontology), how we should study that reality (epistemology) and what study designs and methods we might use (methodology). Social science research can be conceptual or empirical, quantitative or qualitative, and descriptive or analytical – or a combination of all these.

The imperative to include the social sciences in equity oriented studies of health systems and policy research, was illustrated a few years ago by an outstanding review by the Lancet Commission on Culture and Health [1]. David Napier and colleagues distinguished between narrowly biological notions of health and disease and the wider socio-cultural context in which people become sick, illness is experienced and managed, and health services and systems emerge and evolve. This dual perspective is illustrated, for example, by the many ways in which it is possible to study nutrition. As Crotty has pointed out, “The act of swallowing divides nutrition’s ‘two cultures’, the post swallowing world of biology, physiology, biochemistry and pathology, and the pre-swallowing domain of behaviour, culture, society and experience” [2].

More generally, the development, course and outcome of disease (especially in vulnerable groups) is often profoundly shaped by influences requiring a social science research approach: the meso-level environment of the family, school, workplace and community and the macro-level context of social, cultural, political and economic forces. Specific influences include (for example) childcare practices and gender roles; patterns of migration; societal acceptance of, or discrimination towards, particular groups; availability and consumption patterns for food, alcohol and tobacco. So too is social science research central to revealing the nature of health system operations and impact, including consideration of the many domains of accessibility of health services; trust (or lack of it) in public institutions; and political and policy discourses about what causes ill health, how services should be funded and what kinds of care, for whom, should be covered from the public purse.

For many years, social science research has given voice to the oppressed and disadvantaged, as illustrated by Mildred Blaxter’s classic study (using both quantitative and qualitative methods) of people’s own conceptions of the reasons for health inequalities [3] (a study which has been replicated by many subsequent teams and synthesised in a recent meta-ethnography [4]) and Hilary Graham’s qualitative study of the complex interactions between socio-economic status, caring responsibilities and smoking in disadvantaged women [5].

This new series of empirical papers on social science approaches in health systems and policy research was inspired partly by an open letter to the British Medical Journal from over 80 senior academics bemoaning the lost opportunities in health-related qualitative research [6]. Inadequate recognition of the value of social sciences – but particularly qualitative - research in medical and public health journals makes exploration and explanation of the above-described aspects of health system difficult and inhibits the development of the field. This collection is thus a timely reminder from the HPSR community of the need to challenge the (intended or unintended) silencing of non-quantitative, non-positivist research paradigms and evidence, in the broader pursuit of stronger more responsive health systems.

The papers in this series illustrate a number of aspects of the contribution which qualitative social science research can make to the field of health policy and systems research. A common feature of a number of the studies [7–9] is the use of narrative as a synthesising device to pull together multiple structural influences on the behaviour of an individual participant (the case narrative) or the unfolding of a local programme (case study). The use of narrative methods, whether to collect data or to synthesise or illustrate findings, is often an excellent way of capturing the richness of real-world data – though it is not without its challenges [10].

The methodological uniqueness and heterogeneity of the papers is also worth comment. Quality in mainstream biomedical research is defined largely in terms of methods. Particular study designs (notably, the randomised controlled trial) are viewed as inherently superior to other designs [11], and when we do a systematic review, we generally accept (or reject) relevant primary studies on the grounds of *methodological* quality (or flaws) [12]. As a result, quantitative epidemiological research tends to be restricted to a narrow range of “approved” methods. As the papers in this series illustrate, the social sciences are characterised by a much broader and less prescriptive range of methodological approaches, which are typically combined in unique, imaginative and often pragmatic ways to build a rich dataset from which interpretive insights about a human or societal problem might be drawn.

In sum, there is no set method or combination of methods that defines a high-quality social science study. Rather, the emphasis when assessing study quality is on what Judge and Bauld once called “strong theory, flexible methods” [13]. Theory is important because it frames social science research. Are noncommunicable diseases, for example, largely the result of individual behaviour choices (specifically, the preventable risk factors of tobacco and alcohol use, salt intake, and energy imbalance leading to obesity and type 2 diabetes) [14] – or are these diseases ‘caused’ by obesogenic environments, corporate greed or political inertia [15]? Let us not forget that public policy is made of language and that theories are rhetorical devices which foreground particular ways of arranging and interpreting data and render other interpretations less visible or less credible.

Finally, the papers in this series also illustrate, in different ways and to different degrees, the role of *critical* social science – a term introduced by German sociologists in the 1930s which emphasises the role of academic researchers in social critique and emancipation of oppressed groups – in health policy and systems research. Critical social science asks, for example, whose definitions count, who makes the rules and whose voice is not being heard. The term “structural violence” refers to how social conditions can substantially limit the opportunities and capabilities of individuals, particularly the less fortunate, which goes a long way to explaining why the world’s poor are unfairly burdened by disease and the absence of wellbeing [1] and why health systems still so often exacerbate, rather than ameliorate, these vulnerabilities [16]. Methodological approaches in the critical social science genre include participatory and action research methods which emphasise research *with* (as opposed to *on*) marginalised groups and the role of collectively produced knowledge in developing critical consciousness in such groups [17, 18]. Both this special issue, and thematic series to follow, represent an opportunity for health policy and health systems researchers to contribute critically to the production of new insights, knowledge and methods in this fast-evolving field.
